# Survival and clinical outcomes in patients with metastatic epidural spinal cord compression after spinal surgery: a prospective, multicenter, observational cohort study

**DOI:** 10.1186/s40880-016-0091-5

**Published:** 2016-03-16

**Authors:** Anick Nater, Michael G. Fehlings

**Affiliations:** Department of Surgery, Division of Neurosurgery, University of Toronto, Toronto, ON Canada; Division of Neurosurgery, Toronto Western Hospital, University Health Network, 399 Bathurst St., 4W-449, Toronto, ON M5T 2S8 Canada

**Keywords:** Metastatic epidural spinal cord compression, Prospective study, Cohort study, Surgery, Clinical outcomes

## Abstract

**Background:**

High quality studies have been challenging to undertake in patients with metastatic epidural spinal cord compression. Nonetheless, in the article “Survival and Clinical Outcomes in Surgically Treated Patients With Metastatic Epidural Spinal Cord Compression: Results of the Prospective Multicenter AOSpine Study” recently published in the *Journal of Clinical Oncology*, our team provided convincing evidence that spinal surgery improves overall quality of life in patients with this potentially devastating complication of cancer. Considering that metastatic spinal lesions treated with surgery have the highest mean cost among all oncological musculo-skeletal issues, it is essential to provide high quality data to optimize the therapeutic approaches and cost-effective use of health care resources.

**Main body:**

Although the AOSpine Study provided high quality prospective data, it was primarily limited by the lack of non-operative controls and the relatively small sample size. Given the dearth of medical equipoise and the fundamental difference between patients deemed to be adequate surgical candidates and those who are not amenable to operative intervention, conducting a randomized controlled trial in this patient population was not felt to be ethically or medically feasible. Consequently, the optimal option to overcome limitations of both the lack of controls and the relatively small sample size is through collection of large prospective datasets through rigorously developed and maintained registries.

**Conclusions:**

With the alarming increase in the incidence of cancer in China and China’s parallel growing cancer control efforts, China would offer a fantastic platform to set up a national metastatic spinal lesion registry. Such registry would not only enhance metastatic epidural spinal cord compression translational research but also optimize patient care.

## Background

This commentary is particularly timely since February 4th marks each year the World Cancer Day, which promotes global education, detection, and treatment of cancer through hundreds of events and activities organized worldwide.

In today’s scientific and medical climate, regardless of the field, progress faces two main areas of challenge: technical and economical, which often intertwine and influence each other. Although historically a diagnosis of metastatic epidural spinal cord compression (MESCC) was almost equivalent to a rapidly impending death sentence, clinicians endeavoured to optimize the quality of life of these patients. Surgery, in the form of laminectomy, followed by radiotherapy was a prevalent treatment option until the early 1980s [1] when a few retrospective studies [2–4] and one small randomized clinical trial (RCT) [5] reported that radiotherapy alone was effective in alleviating pain and improving both ambulation and autonomic dysfunction. In addition, a prospective study stated that over 50% of MESCC patients developed evidence of vertebral collapse and over 75% suffered a major neurological deterioration after laminectomy [6]. Consequently, medical therapy in terms of corticosteroids and external beam radiotherapy became the therapeutic mainstay for metastatic spinal lesions [2–4]. Oncologists thus retained a primary role in the care of MESCC patients while the involvement of surgeons subsided significantly.

However, a better understanding of the principles of spinal stability and advances in spinal surgical techniques and internal fixation devices allowed the “rebirth” of surgery as an adjunct to conventional medical treatments. In 2005, Patchell et al. [7] conducted a milestone RCT which demonstrated that de novo circumferential decompressive and reconstructive surgery followed by radiotherapy was superior in improving neurological outcomes than radiotherapy alone. In the United States, the number of MESCC patients who underwent surgical treatment increased by nearly 60% in 2009 compared to 2000 [8]. More specifically, Kelly et al. [9]. reported that the rate of spinal surgery rose from an average of 3.8% to 4.9% per metastatic admission per year after the publication of Pathell’s RCT. Considering that of all skeletal-related events occurring in cancer patients, MESCC treated with surgery is currently associated with the highest mean costs [10, 11], evaluating the survival and clinical outcomes in MESCC patients who undergo surgical treatment is a topic of great interest not only for humanistic and clinical reasons but also for socio-economic reasons.

## Main text

In our recent manuscript published in *Journal of Clinical Oncology*, we reported the results of the AOSpine North America MESCC study, a prospective, multicenter, observational, cohort study conducted over a 5-year period in ten North American sites for which a total of 142 surgically treated patients with a single symptomatic MESCC lesion were followed postoperatively for at least 12 months [12]. The goal was to evaluate the impact of surgery on both clinician-assessed criteria and patient-reported functional and health-related quality of life (HRQoL) outcomes using validated instruments, such as the International Standards For Neurologic Classification of Spinal Cord Injury (ISNCSCI), Short Form 36 Health Survey version 2 (SF-36 v2), and EuroQol 5 dimensions (EQ-5D). Although this study provided high quality data given its prospective design and rigorous externally monitored data collection and coding, it was primarily limited by its lack of controls and the small sample size. The lack of controls is a difficult issue to address. Due to serious ethical concerns, conducting a study in which patients suffering from a symptomatic MESCC lesion and deemed adequate surgical candidates would be randomly assigned to either a surgical or a non-surgical treatment arm seems virtually impossible. Moreover, MESCC patients considered adequate surgical candidates are fundamentally different from those who are not considered able to tolerate a spinal operative procedure; comparing these two sub-populations would represent a major threat to internal validity. In this context, the optimal option to overcome limitations of both the lack of controls and small sample size is through using a registry.

Cancer mortalities have been increasing in China for the past 30 years and cancer is now the second leading cause of death [13, 14]. The Agency for Research on Cancer (IARC), the specialized cancer agency of the World Health Organization (WHO), reported that Chinese patients accounted for 21.8% of the total number of newly diagnosed cancer patients in the world, with over 3.06 million of new cases, and 26.9% of cancer death across the globe, with 2.2 million of cancer death cases, in 2012 [15]. Considering that approximately 10% of cancer patients will develop symptomatic spinal metastasis, 50% of which will require treatment, and 5% to 10% of which will involve spinal surgery [16], we could estimate that of those 3.06 million of people diagnosed with cancer, 306,000 developed symptomatic spinal metastasis, 153,000 needed treatment, and up to over 30,000 underwent spinal surgery. Therefore, over two per 100,000 people of the overall Chinese population may have required spinal surgery for a MESCC lesion in 2012. Given that lung cancer is the most common cancer in China [13, 14] and accounts for about 20% of MESCC [17, 18], this could likely underestimate the magnitude of spinal surgeries performed for MESCC in China today. Consequently, with China’s growing effort in fighting not only lung cancer [19] but cancer at large, China would offer a fantastic platform to set up a national MESCC registry.

Data completeness is the most important factor in achieving a high quality registry. Having a designated individual in every cancer institution responsible for thoroughly collecting patient demographics and clinical information as soon as a patient is diagnosed with spinal metastasis would provide data of the utmost value to perform epidemiologic, clinical, and economic analyses. To date, patient’s life expectancy has been given the greatest weight in orienting clinical decision-making processes for MESCC patients. However, maintenance or enhancement of quality of life is also a crucial aspect to consider in this patient population.

## Conclusions

The AOSpine MESCC study provided high quality data suggesting that the majority of MESCC patients suffering from a single symptomatic lesion would have their overall HRQoL improved postoperatively. To build on this work, we would strongly advocate for the development of high quality prospective registries for metastatic spinal cancer. Such registries, including those undertaken by the Global Spine Tumor Study Group (GSTSG) [20] and the AOSpine Knowledge Forum-Oncology [21], would include questions such as: what are the modifiable and non-modifiable preoperative predictive factors of survival, neurological, functional, and HRQoL outcomes, and of complications in surgical MESCC patients or which outcome measure(s) are best suited for these patients.

Such registries would enhance not only MESCC research but also patient care. MESCC patients would be systematically identified as soon as they are referred to an oncologist and could therefore be put under the care of a multidisciplinary team consisting of medical and radiation oncologists, radiologists, and spinal surgeons as part of a standardized protocol management for MESCC patients. This would, for instance, prevent patients with symptomatic MESCC lesions being referred too late to a spinal surgeon and thus would maximize HRQoL outcomes after treatment.

## References

[1] White WA, Patterson RH, Bergland RM (1971). Role of surgery in the treatment of spinal cord compression by metastatic neoplasm. Cancer.

[2] Gilbert RW, Kim JH, Posner JB (1978). Epidural spinal cord compression from metastatic tumor: diagnosis and treatment. Ann Neurol..

[3] Greenberg HS, Kim JH, Posner JB (1980). Epidural spinal cord compression from metastatic tumor: results with a new treatment protocol. Ann Neurol..

[4] Findlay GF (1984). Adverse effects of the management of malignant spinal cord compression. J Neurol Neurosurg Psychiatry.

[5] Young RF, Post EM, King GA (1980). Treatment of spinal epidural metastases. Randomized prospective comparison of laminectomy and radiotherapy. J Neurosurg.

[6] Findlay GF (1987). The role of vertebral body collapse in the management of malignant spinal cord compression. J Neurol Neurosurg Psychiatry.

[7] Patchell RA, Tibbs PA, Regine WF, Payne R, Saris S, Kryscio RJ (2005). Direct decompressive surgical resection in the treatment of spinal cord compression caused by metastatic cancer: a randomised trial. Lancet.

[8] Yoshihara H, Yoneoka D (2014). Trends in the surgical treatment for spinal metastasis and the in-hospital patient outcomes in the United States from 2000 to 2009. Spine J..

[9] Kelly ML, Kshettry VR, Rosenbaum BP, Seicean A, Weil RJ (2014). Effect of a randomized controlled trial on the surgical treatment of spinal metastasis, 2000 through 2010: a population-based cohort study. Cancer.

[10] Jayasekera J, Onukwugha E, Bikov K, Mullins CD, Seal B, Hussain A (2014). The economic burden of skeletal-related events among elderly men with metastatic prostate cancer. Pharmacoeconomics..

[11] Pereira J, Body JJ, Gunther O, Sleeboom H, Hechmati G, Maniadakis N, et al. Cost of skeletal complications from bone metastases in six European countries. J Med Econ. 2016;1–21. doi:10.3111/13696998.2016.1150852. 10.3111/13696998.2016.1150852 26849381

[12] Fehlings MG, Nater A, Tetreault L, Kopjar B, Arnold P, Dekutoski M (2016). Survival and clinical outcomes in surgically treated patients with metastatic epidural spinal cord compression: results of the prospective multicenter aospine study. J Clin Oncol.

[13] Zhao P, Dai M, Chen W, Li N (2010). Cancer trends in China. Jpn J Clin Oncol.

[14] Chen W, Zheng R, Zeng H, Zhang S (2015). The updated incidences and mortalities of major cancers in China, 2011. Chin J Cancer..

[15] International Agency for Research on Cancer (2016). Globocan 2012: estimated cancer incidence, mortality and prevalence worldwide in 2012.

[16] Sciubba DM, Petteys RJ, Dekutoski MB, Fisher CG, Fehlings MG, Ondra SL (2010). Diagnosis and management of metastatic spine disease: a review. J Neurosurg Spine..

[17] Prasad D, Schiff D (2005). Malignant spinal-cord compression. Lancet Oncol..

[18] Morgen SS, Lund-Andersen C, Larsen CF, Engelholm SA, Dahl B (2013). Prognosis in patients with symptomatic metastatic spinal cord compression: survival in different cancer diagnosis in a cohort of 2321 patients. Spine(Phila Pa 1976)..

[19] Yan L, Xu L (2015). Global efforts in conquering lung cancer in China. Chin J Cancer..

[20] Choi D, Morris S, Crockard A, Albert T, Bunger C, Fehlings M (2013). Assessment of quality of life after surgery for spinal metastases: position statement of the global spine tumour study group. World Neurosurg..

[21] AOSpine. Aospine knowledge forums. Davos, Switzerland. Available at: https://aospine.aofoundation.org/Structure/research/KnowledgeForum/Pages/knowledge-forum.aspx.

